# Intramuscular Priming and Intranasal Boosting Induce Strong Genital Immunity Through Secretory IgA in Minipigs Infected with *Chlamydia trachomatis*

**DOI:** 10.3389/fimmu.2015.00628

**Published:** 2015-12-16

**Authors:** Emma Lorenzen, Frank Follmann, Sarah Bøje, Karin Erneholm, Anja Weinreich Olsen, Jørgen Steen Agerholm, Gregers Jungersen, Peter Andersen

**Affiliations:** ^1^Section for Veterinary Reproduction and Obstetrics, Department of Large Animal Sciences, Faculty of Health and Medical Sciences, University of Copenhagen, Copenhagen, Denmark; ^2^Department of Infectious Disease Immunology, Chlamydia Vaccine Research, Statens Serum Institut, Copenhagen, Denmark; ^3^Section for Immunology and Vaccinology, National Veterinary Institute, Technical University of Denmark, Copenhagen, Denmark

**Keywords:** *Chlamydia trachomatis*, minipig model, vaccine, vaccination strategy, mucosal vaccination/immunization, intranasal immunization, mucosal immunity

## Abstract

International efforts in developing a vaccine against *Chlamydia trachomatis* have highlighted the need for novel immunization strategies for the induction of genital immunity. In this study, we evaluated an intramuscular (IM) prime/intranasal boost vaccination strategy in a Göttingen Minipig model with a reproductive system very similar to humans. The vaccine was composed of *C. trachomatis* subunit antigens formulated in the Th1/Th17 promoting CAF01 adjuvant. IM priming immunizations with CAF01 induced a significant cell-mediated interferon gamma and interleukin 17A response and a significant systemic high-titered neutralizing IgG response. Following genital challenge, intranasally boosted groups mounted an accelerated, highly significant genital IgA response that correlated with enhanced bacterial clearance on day 3 post infection. By detecting antigen-specific secretory component (SC), we showed that the genital IgA was locally produced in the genital mucosa. The highly significant inverse correlation between the vaginal IgA SC response and the chlamydial load suggests that IgA in the minipig model is involved in protection against *C. trachomatis*. This is important both for our understanding of protective immunity and future vaccination strategies against *C. trachomatis* and genital pathogens in general.

## Introduction

Most of all human infections are established at the mucosal surfaces (1), including sexually transmitted infections with *Chlamydia trachomatis*, the most common sexually transmitted bacterium globally. *C. trachomatis* is a major global health problem causing more than 100 million new cases of genital chlamydia each year (2). Even though the infection can be treated with antibiotics, the frequent asymptomatic course of infection, with up to 75% symptom-free infections, makes the infection difficult to combat. Untreated infections can cause severe permanent complications, such as pelvic inflammatory disease, ectopic pregnancy, and infertility in women (3).

Screening programs and treatments have been intensified to lower the prevalence of *C. trachomatis* infections, largely without the expected impact on the incidence of *C. trachomatis* cases. Therefore, large international efforts are focused on the development of a vaccine (3–6).

*Chlamydia trachomatis* enters the body through the mucosal membrane in the genital tract and has a complex lifestyle that has been a significant challenge for the design of a vaccine (7). Initial bacterial control is most efficiently mediated through mucosal neutralizing antibodies (8–11), but interferon gamma (IFN-γ) producing Th1 cells becomes pivotal for protection as the bacteria infects the epithelial layer and localize intracellularly (3, 4, 8). Traditional intramuscular (IM) vaccination strategies have recently been implemented against the genital human papillomavirus and the correlate for efficacy is specific neutralizing systemic IgG (12–14). Whether the same strategy can be employed for *C. trachomatis* is currently unclear but in terms of neutralizing antibodies, secretory IgA (SIgA) provide a significant theoretical advantage in *C. trachomatis* immunity due to its anti-inflammatory capacity compared to monomeric IgA and IgG (15, 16). The hypothetical advantage of anti-inflammatory antibodies is to avoid excessive inflammation and thereby immune-mediated pathology. Thus, vaccination protocols for the induction or redirection of mucosal responses, i.e., SIgA, are the subject of intense research (4, 17).

Most mucosal compartments of the body have local mucosal immune inductive sites such as gut-associated lymphoid tissue and nasal-associated lymphoid tissue. The associated lymphoid tissues are responsible for the induction of mucosal immunity in the respective mucosal compartments (18, 19). However, as the genital tract lacks these immune inductive sites (18, 20), it is important to develop an alternative immunization strategy that utilizes other mucosal inductive sites to promote local genital tract immunity. It has been reported that intranasal (IN) immunization can induce mucosal immunity in both the respiratory and the genital tracts (1, 21–24). Recently, IL-17 secreting CD4^+^ T-helper cells (Th17 cells) have been recognized as a key component in the acceleration of mucosal immunity and IgA secretion (25, 26) and studies in mice have shown that prime-boost regimes that includes a Th17 prime is superior for the induction of mucosal IgA (Christensen et al., unpublished).

However, studies in mice can be difficult to translate into man as the murine hormonal cycle, reproductive organs, and some parameters within the immune system differ significantly from humans (27). It is therefore important to verify murine concepts in animal models that resemble the human organ system of interest. Non-human primates (NHPs) offer the closest resemblance of humans, however ethical and practical concerns make it difficult to perform experiments in NHPs. Pigs offer a great alternative, by having a reproductive cycle, genital tract, and immune system that resemble those of humans to a high degree (28) and therefore may have better predictive value in preclinical evaluation of novel vaccination strategies for genital tract immunity.

With the overall aim to develop an immunization protocol for the induction of local genital immunity against *C. trachomatis*, we have evaluated the potential of IM prime followed by IN boost in the minipig model. We used a multisubunit vaccine with recombinant *C. trachomatis* antigen formulated with CAF01 (29), an adjuvant reported to induce a Th1/Th17 response together with high antibody titers (8, 30). Our study demonstrates that IN boosting in IM/CAF01 primed minipigs induces a striking local IgA immune response in the genital tract and an accelerated clearance of genital *C. trachomatis* infection.

## Materials and Methods

### *Chlamydia* *trachomatis*

*Chlamydia trachomatis* serovar D (*Ct* SvD; UW-3/Cx, ATCC^®^ VR-885™), originally isolated from the cervix of a female patient with an asymptomatic infection, was grown in HeLa-229 cells, harvested, and purified as previously described (31, 32).

### Vaccine and Adjuvant

Statens Serum Institut (Copenhagen, Denmark) was the provider of *C. trachomatis* vaccine antigen and adjuvant. The minipigs were vaccinated with a hybrid vaccine consisting of two recombinant *C. trachomatis* fusion proteins designated Hirep1 (8) and CTH93 formulated with CAF01 (Cationic Adjuvant Formulation 01) adjuvant (29). The Hirep1 subunit is composed of repeated B cell epitopes with VD4 regions of MOMP from SvD, SvE, and SvF (8). The CTH93 subunit has three T cell epitopes included and is composed of CT043 in full length, CT414_aa605-804_, and MOMP SvD_aa34-371_ (33).

The vaccines for IM administration were composed of 5 μg Hirep1 and 5 μg CTH93 (in total 10 μg antigen) in 1 ml Tris buffer (10 nM, pH 7.4) mixed with 1 ml CAF01 and sucrose to a 9% isotonic solution. The vaccines for IN administration were composed of 100 μg Hirep1 and 100 μg CTH93 in 0.5 ml Tris buffer and either 0.5 ml 2 × CAF01 (adjuvanted) or 0.5 ml Tris buffer (unadjuvanted) plus 9% sucrose.

### Minipigs

The study was performed with 24 specific pathogen-free (http://minipigs.dk/the-goettingen-minipig/health-status/) sexually mature female Göttingen Minipigs (Ellegaard Göttingen Minipigs A/S, Dalmose, Denmark). The minipigs were 5–6 months old at arrival; were housed in isolation units; had 1 week of acclimatization before the start of the study; and were under all circumstances treated in accordance with the Danish law on animal experiments. They were randomly divided into four groups with six animals in each (*n* = 6). The experiment was approved by the Danish Animal Experiments Inspectorate (license number: 2013-15-2934-00978).

### Immunization

**Figure 1:**
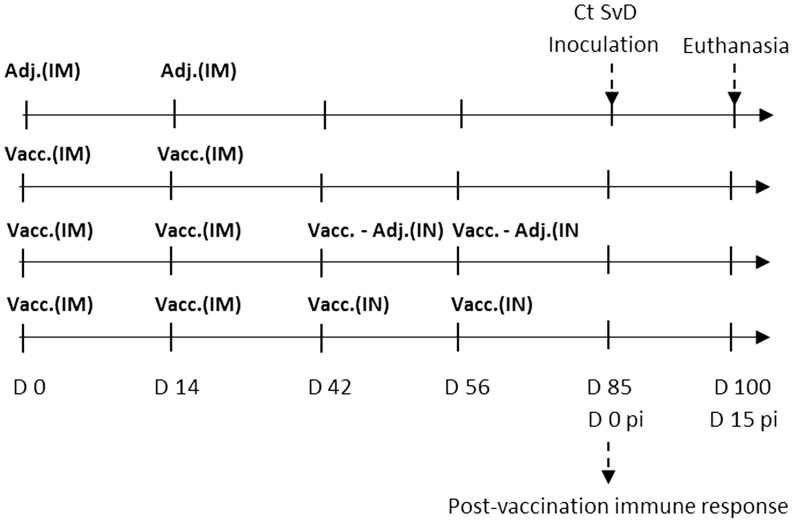
**Experimental setup**. Sexually mature Göttingen Minipigs (*n* = 24) were randomly assigned to four groups with six minipigs in each. The control group was given the adjuvant CAF01 intramuscularly two times (2*Adj.). The three vaccinated groups were vaccinated two times intramuscularly (IM) with the vaccine composed of Hirep1 and CTH93 with CAF01. One group was not boosted (2*IM), one group was given intranasal (IN) boosters with vaccine antigens alone without adjuvant (2*IM/2*IN-Adj.), and the last group was given IN boosters with the complete vaccine including adjuvant (2*IM/2*IN). After the immunizations, the minipigs were estrus synchronized and at day 85 all pigs were challenged with 5 × 10^9^*C. trachomatis* serovar D (Ct SvD) IFUs. Vaginal swabs were collected at days 3, 5, and 7 post infection (pi), and they were euthanized at day 15 pi. Abbreviations: Vacc., vaccine (composed of Hirep1 and CTH93 formulated with CAF01); Adj., adjuvant (CAF01); IM, intramuscular, IN, intranasal.

The IM vaccination was performed in the thigh (*m. biceps femoris*) with a 21-G needle after cleaning the injection area with 70% ethanol. The IN vaccination was performed with a nasal delivery device (LMA MAD Nasal™, Needle-Free Intranasal Drug Delivery, LMA) connected to a 3-ml syringe. The immunization protocol for the four groups is illustrated in Figure 1.

### Experimental Challenge Infection

Following the immunizations, the minipigs were treated with Regumate^®^ (20 mg/minipig/day, orally for 18 days) starting at day 62 after the first immunization, to synchronize their estrous cycle. They were fed individually in this period, to make sure that each minipig had a complete dose each day. Following the Regumate^®^ treatment, the minipigs were monitored for estrous signs (behavior, hyperemia, and swollenness of the vulva). At the time of estrous, the pigs were anesthetized with Zoletil^®^ mixture [1 vial Zoletil^®^50 Vet (125 mg tiletamin and 125 mg zolazepam), 6.25 ml xylazine (20 mg/ml), 1.25 ml ketamine (100 mg/ml), and 2.5 ml butorphanol (10 mg/ml)] (34) and inoculated through an artificial insemination catheter (Osiris, E-vet, Denmark) vaginocervically with 5 × 10^9^*C. trachomatis* SvD inclusion forming units (IFUs). The bacteria were diluted in 2 ml sterile SPG buffer (0.2 M sucrose, 20 mM sodium phosphate, and 5 mM glutamic acid buffer). Following the inoculation, the minipigs were placed with the hindquarters elevated for 20 min to avoid immediate reflux.

### Sampling and Sample Processing

During the immunization period the pigs had their rectal temperature taken 24 and 48 h after each immunization. All animals were monitored daily for clinical signs, and after the challenge they had their rectal temperature taken daily until day 7 post infection (pi) and then again at day 10 pi.

During sampling, the animals were anesthetized by IM injection with Zoletil^®^ mixture (34) (1 ml/17 kg for vaginal swabbing, 1 ml/15 kg for blood collection and vaginal swabbing, and 1 ml/12 kg for inoculation/infection).

Blood samples were collected from *v. Jugularis externa* at the following days after the first immunization: days 0, 14, 42, 56, 85 (0 pi), 92 (7 pi), and 100 (15 pi) with a vacutainer system. Blood samples were collected in coagulation activator-coated tubes for serum isolation and heparin stabilized tubes for isolation of peripheral blood monomuclear cells (PBMCs). Blood samples for serum isolation were centrifuged for 15 min at 3500 rpm, where after the serum was isolated and stored at −20°C.

Vaginal swabs were collected with sterile regular size cotton swabs at the following days after the first immunization: days 0, 14, 42, 56, 85 (0 pi), 88 (3 pi), 90 (5 pi), 92 (7 pi), and 100 (15 pi) for determination of mucosal antibody titers and *C. trachomatis* detection. All vaginal swabs were collected in 0.5 ml SPG and taken with the help from a vaginoscope, aiming for aseptic conditions.

Nasal swabs were taken with sterile minitip cotton swabs. They were moistened with sterile SPG and introduced carefully into the nares following the upper-medial border, rolled on the nasal mucosa and thereafter stored in 0.5 ml SPG.

The vaginal, genital, and nasal swabs were vortexed for 1 min with three sterile glass-beads. The SPG with swab material was stored at −80°C until further analyses.

The minipigs were euthanized 15 days pi by IM injection of 1 ml/7 kg Zoletil^®^ mixture (34) followed by exsanguination. During necropsy, tissue samples and swabs were taken from the cervix, uterine body, uterine horns (bilaterally sampled both proximally and distally), and the uterine tubes (Fallopian tubes). Regular size swabs were used for all swabs except the uterine tubes, where minitip size was used. The iliosacral and retropharyngeal lymph nodes were collected. The lymph nodes were cut into smaller pieces and forced through a metal mesh to obtain a homogenized single-cell suspension. They were washed twice in RPMI-1640 (Gibco, Invitrogen).

### Antibody ELISA

An indirect enzyme linked immunosorbent assay (ELISA) was used to evaluate the antigen-specific antibodies in serum and swab samples. Maxisorp^®^ plates (NUNC A/S, Roskilde, Denmark) were coated with the vaccine antigens Hirep1 and CTH93 (1 μg/ml) over night at 4°C. The isotypes IgG and IgA were detected with HRP-conjugated antibodies specific against the porcine IgG (Goat anti-pig IgG·Fc, 1:10.000, AAI41P, Serotec, UK) and IgA (Goat anti-pig IgA, 1:2000, AAI40P, Serotec, UK). The secretory component (SC) was detected with a mouse anti-pig Ig SC antibody (MCA634, Serotec, UK) followed by a HRP-conjugated goat anti-mouse IgG (H/L) HRP (STAR117P, Serotec, UK). The reactions were visualized with TMB PLUS substrate (KemEnTec, Taastrup, Denmark) and stopped with 0.5 M sulfuric acid. The plates were read on an ELISA reader at 450 nm with correction at 650 nm. Positive serum from a previous study (33) was included as a positive control and two wells were run without substrate as a negative control on each plate. The positive control was used as an internal standard to correct for plate-to-plate variation. The antibody titers were calculated as the reciprocal of the highest dilution with an optical density (OD) value higher than the cutoff. For serum and vaginal swab samples, the cutoff was determined from the day 0 sample mean + 1,745 × SD (35), and in the genital swab samples, the cutoff was determined from the negative control (PBS·T·C) mean + 1,745 × SD (35).

### Cytokine Expression by PBMC and Lymph Node Cells

Peripheral blood monomuclear cells were isolated on a density gradient with Lympholyte^®^ Mammal Cedarlane (CL5110, TriChem Aps, Skanderborg, Denmark) according to the manufacturer’s instructions. PBMC and lymph node cell cultures were made in Nunclon round-bottom 96-well plates (NUNC A/S, Roskilde, Denmark) with 2 × 10^5^ cell/well in 200 μl RPMI-1640 supplemented with 5 × 10^−5^ M 2-mercaptoethanol, 1 mM glutamine, 1% pyruvate, 1% penicillin–streptomycin, 1% HEPES, and 10% fetal calf serum (Invitrogen, Taastrup, Denmark). The cell cultures were re-stimulated in triplicates with the vaccine antigens Hirep1 and CTH93 in 1 μg/ml at 37°C and 5% CO_2_. Stimulation with SEB antigen was used as positive control for cell viability, and media was used as negative control. The cell culture supernatant was collected after 72 h of incubation and stored at −20°C until further analysis. Cytokine concentration in the cell culture supernatants was determined by ELISA. IL-17A was detected with a rabbit anti-pig IL-17A polyclonal-coating antibody (KP0498S-100, Kingfischer Biotech, Saint Paul) (1 μg/ml) biotinylated rabbit anti-pig IL-17A polyclonal detection antibody (KPB0499S-050, Kingfisher Biotech, Saint Paul) in 0.1 μg/ml and HRP-conjugated streptavidin (SNN1004, Life Technologies™, Denmark) in 0.1 μg/ml. IFN-γ was detected with a mouse monoclonal anti-pig IFN gamma-coating antibody (clone P2F6, Thermo Fisher Scientific, Denmark) 1:550 and a biotinylated mouse anti-pig IFN-γ detection antibody (clone P2C11, BD Biosciences, Albertslund, Denmark) 1:500 followed by HRP-conjugated streptavidin (SNN1004, Invitrogen, Taastrup, Denmark) 0.1 μg/ml (36). On each plate, a standard with a known concentration of porcine IFN-γ was added in duplicate serial dilutions together with two wells without substrate as a negative control. A log–log transformed standard curve was made from the serial dilutions of the standard, and an equation was made correlating the measured OD value with the amount of IFN-γ in the sample. This equation was used for determining IFN-γ concentration in the samples.

### *In vitro* Neutralization Assay

An *in vitro* neutralization assay using serum from day 85 was performed to evaluate the protective capacity of the vaccine-induced antibodies. The assay principally followed the protocol described in (37). The secondary antibody (Alexa Flour 488, goat-anti-rabbit IgG, A11008, Life Technologies) was added in the dilution 1:500. The evaluation in the fluorescence microscope was performed by quantifying the number of inclusions in 20 fields of view at 40× magnification (Olympus IX71 inverted microscope). Neutralization was calculated as the percentage reduction in the number of IFU compared to the sera from control group animals (2*Adj.).

### qPCR Detection of *C. trachomatis* on the Vaginal Mucosa

DNA extraction from the vaginal swab samples was performed with Chelex^®^100 (Bio-Rad, Life Science, Denmark). The 100 μl of the swab material was mixed with 300 μl of a 20% Chelex solution in TE buffer (T9285, Sigma Aldrich), vortexed for 60 s, and incubated at 96°C for 10 min. The sample was then centrifuged for 10 min at 17,500 g and 4°C and hereafter triplicates of 5 μl of the supernatant was used for PCR.

Real-time qPCR detection of *C. trachomatis* in the vaginal swab samples was performed by detection of the 16S rRNA gene. Internal control (IC) and *C. trachomatis* (*Ct*) 16s primers were bought from TAG Copenhagen A/S (Copenhagen, Denmark) with the following sequence: IC-F 5′ACCGCTCAGGCATTTGCT-3′, IC-R 5′CCGGGACGTATCATGCT3′, Ct 16s-F GGATCTTCGGACCTTTCGGT, Ct 16s-R AATCTCTCAATCCGCCTAGACA. The probes were bought from Applied Biosystems (Life Technologies Europe BV, Naerum, Denmark) with the following sequence: Ct 16s-probe FAM-AAGGGAGAGTCTATGTGATAT – MGBNFQ and IC-probe NED-TCCTTCGTGATATCGGACGTTGGCTG – MGBNFQ.

The assay was performed with a final reaction volume of 25 μl with Perfecta qPCR SuperMix [UNG, low ROX, 95066-02K (2000 rx) Quantum Biosciences, Gaithersburg, MD, USA], 300 nM of each primer; Ct 16s-F, Ct 16s-R, IC-F, and IC-R and 75 nM of each of the Ct 16s-probe and IC-probe, IC-DNA and DEPC treated water was added up to a total volume of 20 μl. MicroAmp Fast 96-well plates (Applied Biosystems, Naerum, Denmark) were used with an adhesive cover (MicroAmp Optical Adhesive film, Applied Biosystems, Denmark).

The samples were run on a StepOne™ Real-time PCR instrument (Applied Biosystems^®^), and the instrument was programed to run 2 min at 95°C and 40 cycles of denaturation at 95°C for 15 s and annealing/extension at 60°C for 1 min. Based on the negative control, the *C*_t_ cutoff was determined to be 36, hence *C*_t_ values >36 were considered as nonsense.

### Statistical Analysis

Enzyme linked immunosorbent assay results were normalized according to the IC on each plate. Gaussian distribution of all data was analyzed by D'Agostino and Pearson omnibus normality test in Graph Pad Prism 5 (GraphPad Software Inc., CA, USA). All statistics were performed with GraphPad Prism. The not-normally distributed data were if possible log transformed and analyzed with one-way ANOVA followed by Bonferroni corrected multiple comparisons. If log transformation was not possible, they were analyzed with non-parametric tests; Kruskal–Wallis test and Dunns multiple comparison test. Analysis of correlation was performed with Spearman’s rank correlation coefficient. The group wise results are presented with mean ± SEM errorbars (38). The comparisons were considered statistical significant if the *P* value was <0.05 (*P* < 0.05). Further levels of significance are indicated with asterisks *P < 0.05, **P < 0.01, ***P < 0.001.

## Results

### Experimental Design

To evaluate the effect of a combined vaccination strategy based on systemic priming and mucosal boosting, groups of Göttingen Minipigs were immunized using different IM prime/IN boost regimes and subsequently given a vaginocervical *Ct* SvD infection. The vaccine was a cocktail of two recently developed recombinant vaccine fusion proteins, Hirep1 and CTH93 (8, 33) formulated with the CAF01 adjuvant promoting both cell-mediated immune (CMI) and humoral immune responses (29). Three groups of minipigs were vaccinated and compared to a control group receiving CAF01 adjuvant two times IM (2*Adj.). All three vaccinated groups received two IM vaccinations; either alone (2*IM), boosted with two IN vaccinations (2*IM/2*IN), or boosted IN with proteins alone without adjuvant (2*IM/2*IN-Adj.) (Figure 1). Blood and vaginal swab samples were collected and humoral as well as CMI responses were evaluated before and after challenge infection together with the vaginal *C. trachomatis* load pi.

### Systemic and Mucosal Antibody Responses Post Vaccination

**Figure 2:**
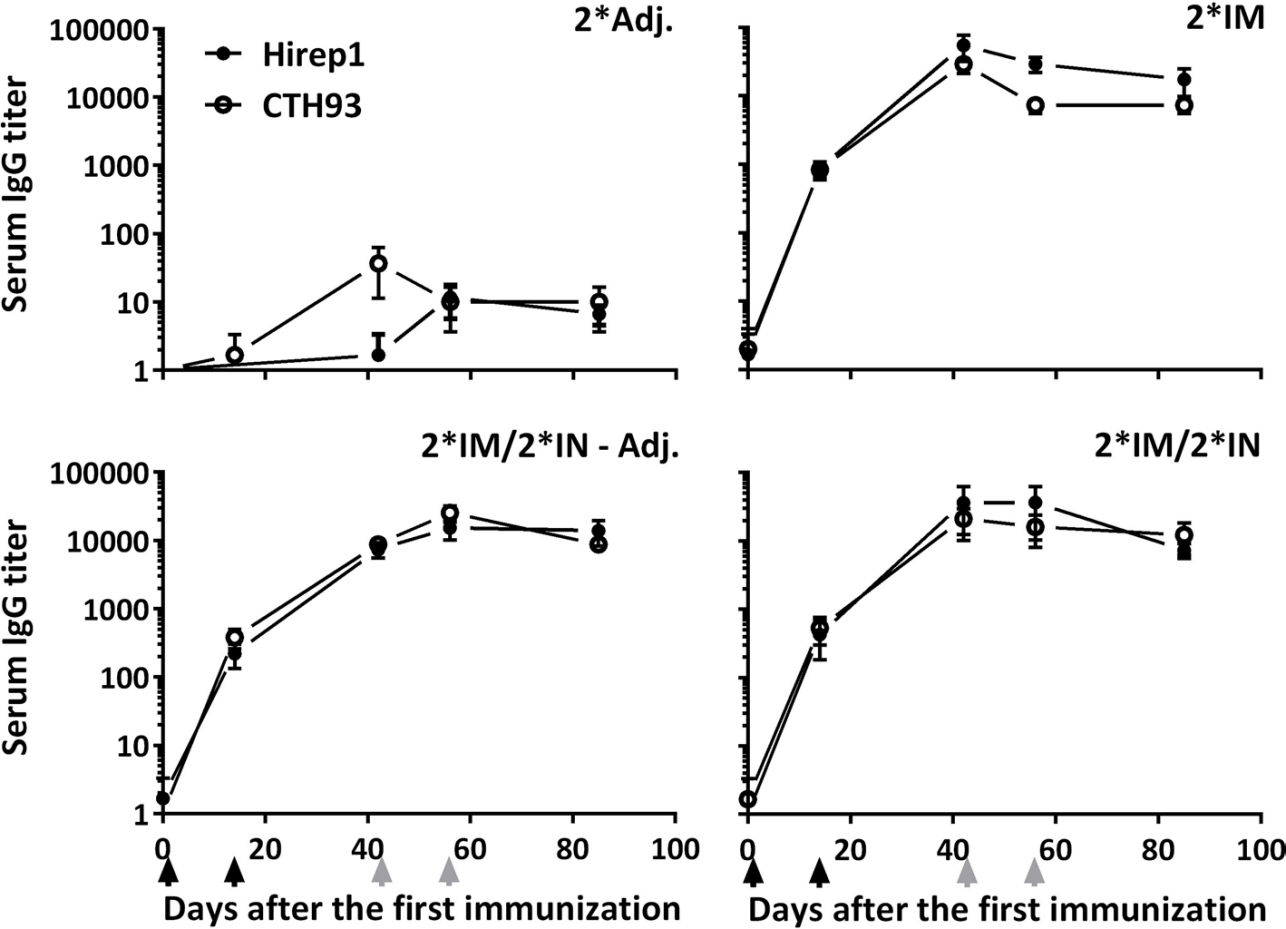
**The kinetics of antigen-specific IgG in serum of vaccinated minipigs**. Serum was collected at various timepoints during the immunization period and evaluated for Hirep1- and CTH93-specific IgG responses by ELISA. Graphs show mean ± SEM. The black arrows indicate the two IM immunizations and the two gray arrows indicate the two IN immunizations.

The level and kinetics of the antibody responses specific for the two vaccine antigens Hirep1 and CTH93 were evaluated in serum and locally on the genital mucosa by ELISA. All vaccinated animals had rapidly increasing titers against both Hirep1 and CTH93 reaching a significant level at day 14 and a stable plateau from days 40 to 85 after the first immunization (Figure 2). Hirep1 and CTH93 promoted equal antibody levels with almost identical kinetics (Figure 2).

**Figure 3:**
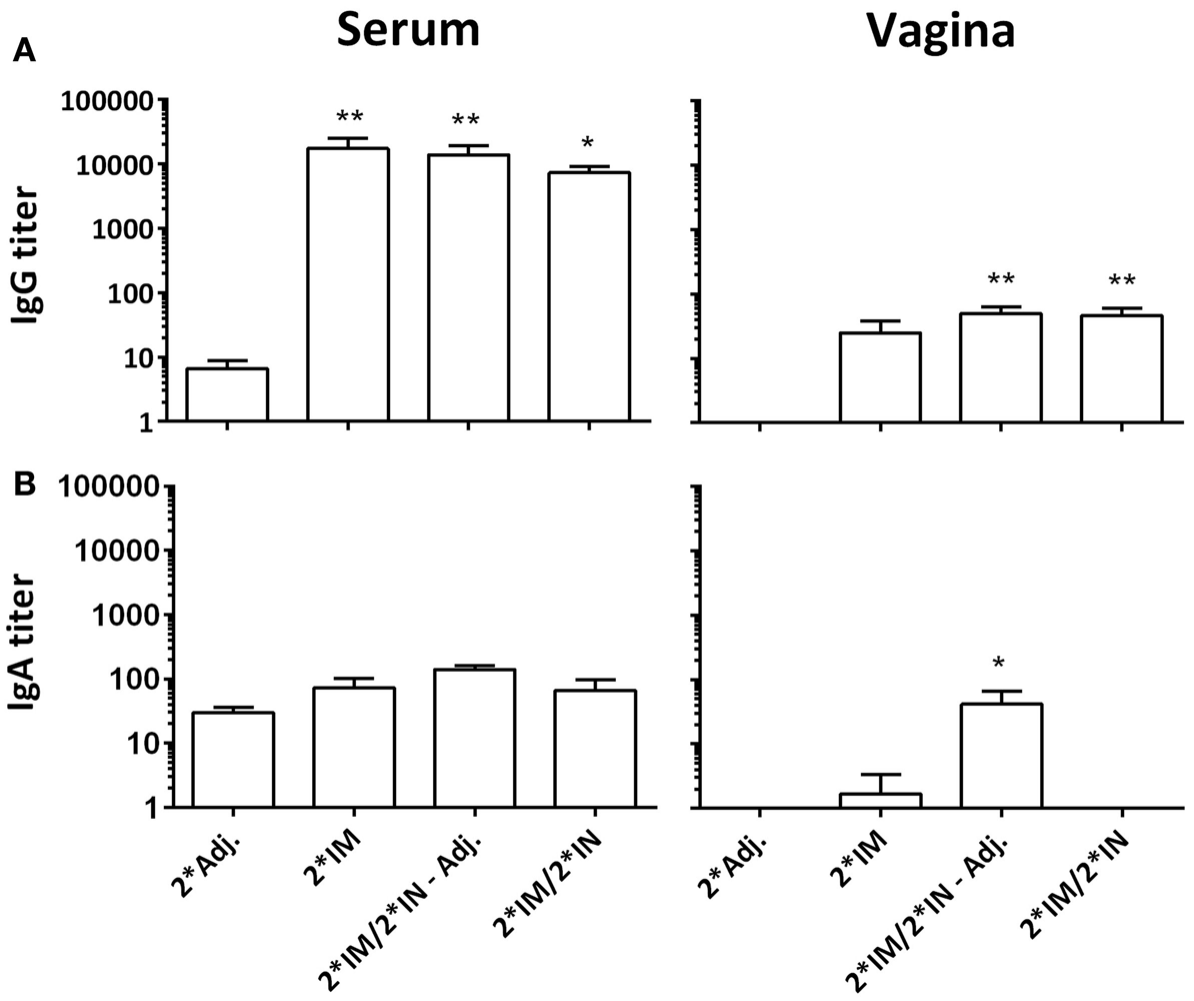
**Hirep1-specific antibody responses in serum and on the vaginal mucosa following the immunizations with Hirep1 and CTH93 as described in Figure 1**. Serum and vaginal swabs from day 85 after the first immunization were assayed for antigen-specific IgG and IgA responses by ELISA. **(A)** Hirep1-specific IgG in serum and vagina. **(B)** Hirep1 specific IgA in serum and vagina. The response against CTH93 was almost identical and therefore not shown. Bars show mean ± SEM. Statistics: Dunn’s multiple comparisons. Asterisks (*) indicate significance compared to the 2*Adj. group.

Following vaccinations, highly significant serum IgG titers were detected in all vaccinated groups (Figure 3), which also led to slightly increased vaginal IgG titers (Figure 3). We detected no difference in serum IgA levels, but in the vaginal samples, we found an increased IgA titer in the group receiving un-adjuvanted IN booster immunizations (Figure 3). In addition, a significant IgA response was found on the nasal mucosa in both IN-boosted groups (mean Hirep1-specific nasal IgA titer: 2*Adj.: 1.7, 2*IM: 18.3, 2*IM/2*IN-Adj.: 18630***, 2*IM/2*IN: 4560*, Dunn’s multiple comparison with the 2*Adj. group).

**Figure 4:**
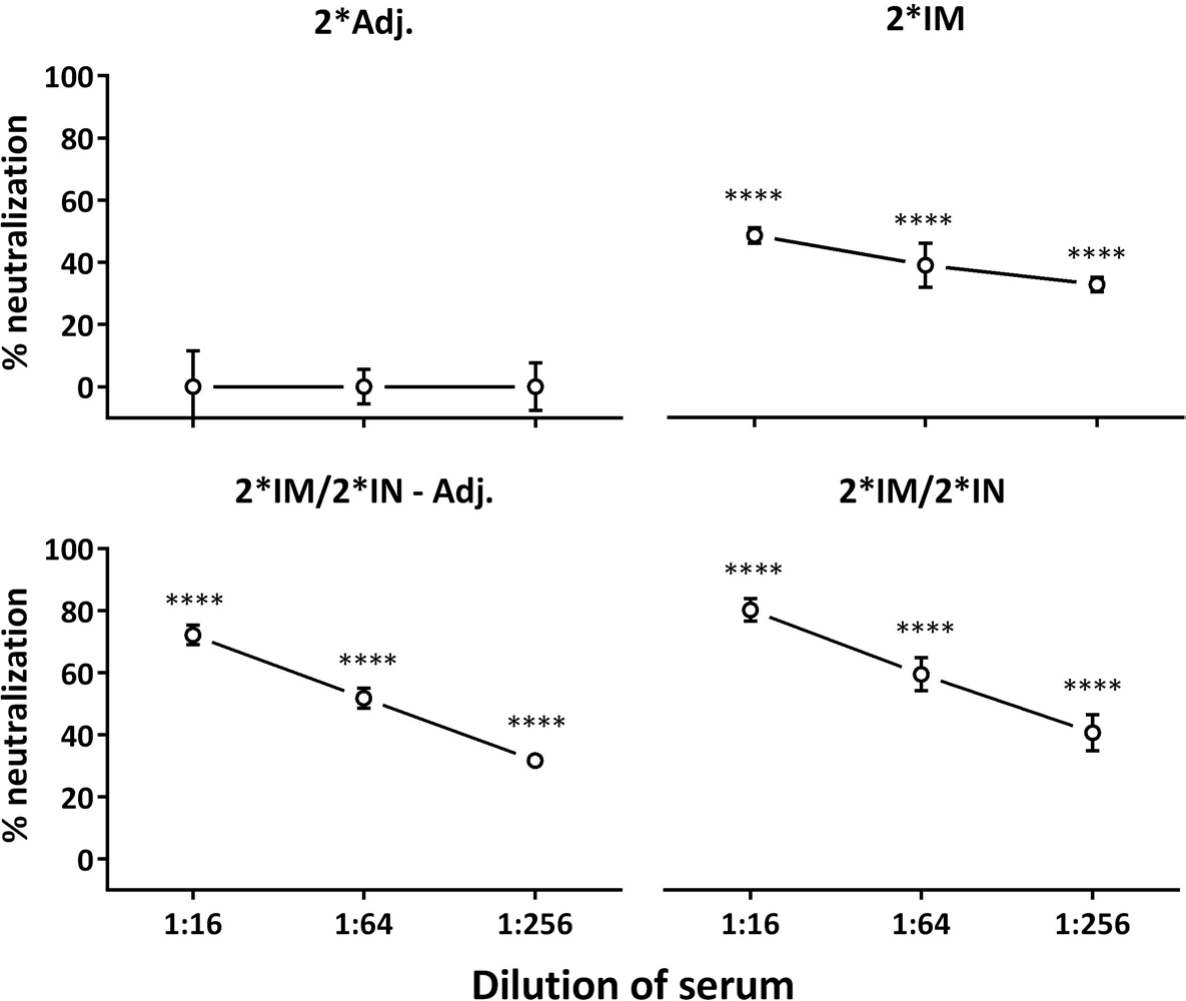
***In vitro* serum neutralization after the immunization period**. Serum from day 85 after the first immunization and *C. trachomatis* SvD EBs were incubated with HaK cells and the ability of serum antibodies from vaccinated minipigs to inhibit *in vitro* chlamydial infection of cells, relative to serum from the 2*Adj. control group, was evaluated. Percent neutralization is given for each group at the three dilutions of serum. Graph shows mean ± SEM. Statistics: Dunn’s multiple comparisons. Asterisks indicate significance compared to the 2*Adj. group.

The vaccine-induced serum antibodies were further evaluated in an *in vitro* neutralization assay. Sera from all three vaccinated groups showed significant neutralization of *C. trachomatis* SvD compared to the 2*Adj. serum (Figure 4). Furthermore, at a serum dilution of 1:16, the two IN-boosted groups showed a significantly higher neutralization than the 2*IM immunized group (Figure 4, 2*IM/2*IN-Adj.: *P* < 0.05, 2*IM/2*IN: *P* < 0.01).

### Cell-Mediated Immune Response Post Vaccination

In order to characterize the vaccine-induced CMI response, the secretion of IFN-γ and interleukin 17A (IL-17A) was evaluated in the supernatants of PBMCs re-stimulated with vaccine antigens, Hirep1 and CTH93.

**Figure 5:**
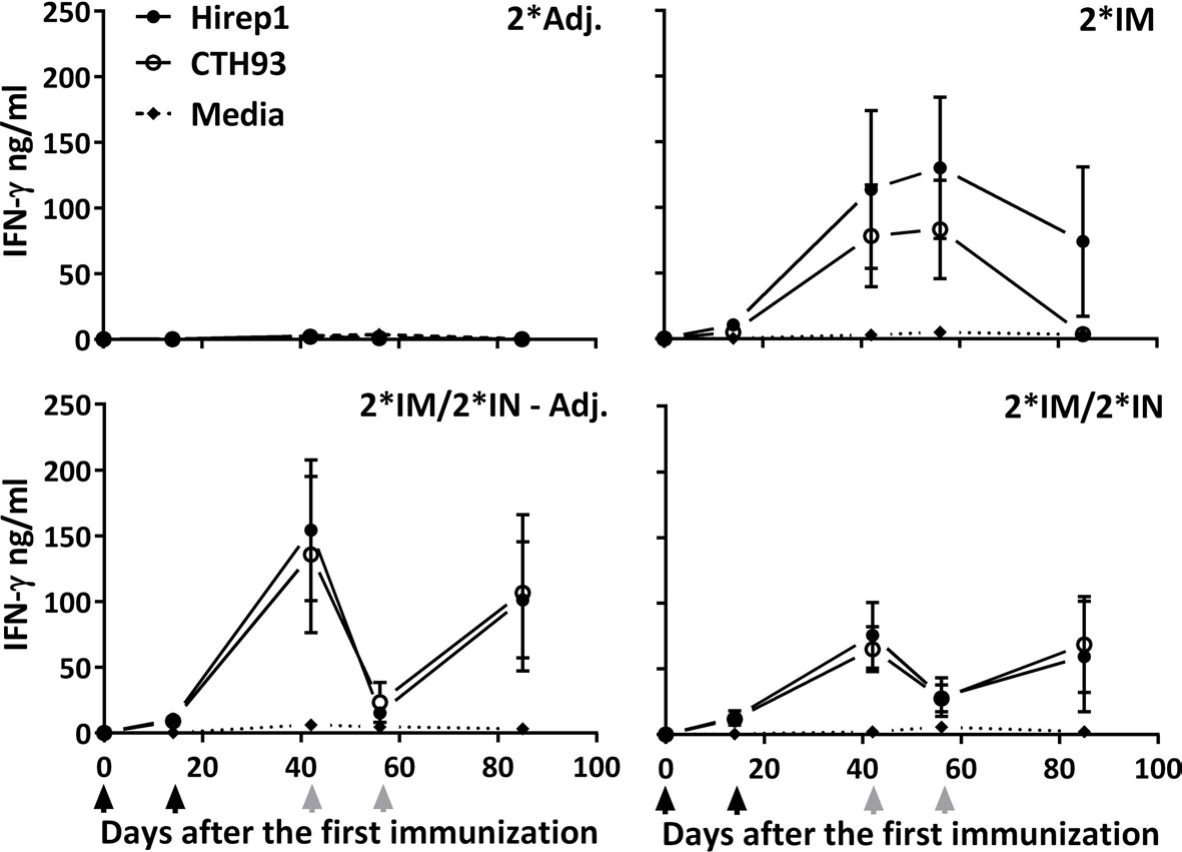
**Cell-mediated IFN-γ response during the immunization period**. The groups of minipigs were immunized according to Figure 1 with Hirep1 and CTH93, and the cell-mediated immune response was evaluated during the immunization period by isolating PBMCs, re-stimulating them with Hirep1, CTH93, and media whereafter the IFN-γ levels were determined in the supernatant. Graphs show mean ± SEM. The black arrows indicate the two IM immunizations and the two gray arrows indicate the two IN immunizations.

All three vaccinated groups raised powerful T cell responses measured by secreted IFN-γ to both vaccine components (Hirep1 and CTH93) with very similar kinetics (Figure 5). The kinetics of the IFN-γ response showed a different pattern in the IN-boosted groups compared to the 2*IM group, with the IN-boosted groups showing a decrease in IFN-γ level after the first IN booster (day 56) (Figure 5). All three vaccinated groups raised a low but significant Hirep1 PBMC IL-17A response at day 85 (2*IM: 141.6 pg/ml****, 2*IM/2*IN – Adj.: 91.9 pg/ml****, and 2*IM/2*IN: 76.5 pg/ml**** compared to the 2*Adj.:3.27 pg/ml).

### Immune Response Post Challenge

**Figure 6:**
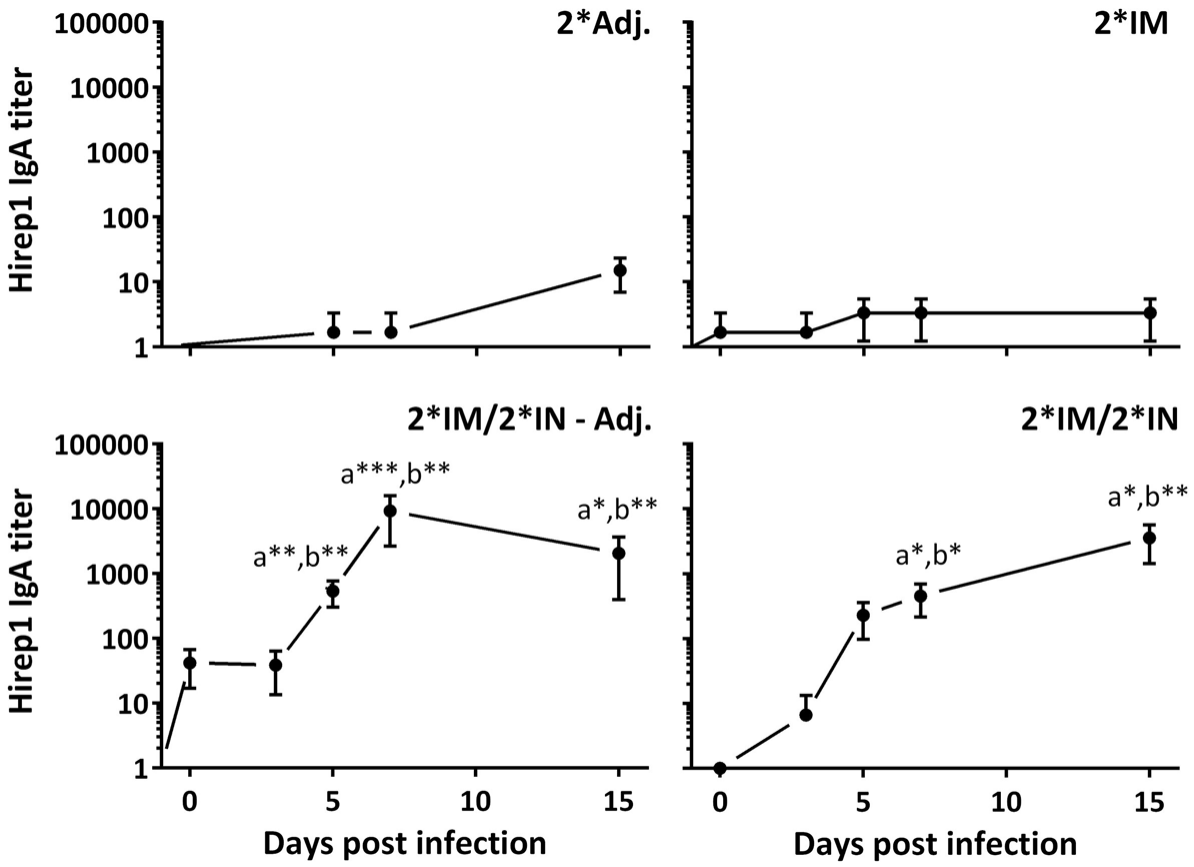
**Vaginal Hirep1-specific IgA response following vaginal challenge**. After the immunization protocol (Figure 1), the minipigs were challenged vaginally with *C. trachomatis* SvD and vaginal swabs were collected and assayed for Hirep1- and CTH93-specific IgA by ELISA. The response against CTH93 was almost identical and therefore not shown. Graphs show mean ± SEM. Statistics: Dunn’s multiple comparisons. Symbol (a) indicates significance compared to the 2*Adj. group and (b) indicates significance compared to the 2*IM group. Asterisks indicate significance level.

The vaccinated minipigs were infected with *C. trachomatis* SvD at day 85 after the first immunization. The inoculation was performed during estrus, and the local as well as the systemic humoral responses were followed for 15 days pi. The two IN-boosted groups showed a significant vaginal IgA response from day 5 pi and for the rest of the study period (Figure 6). The vaginal IgA titer peaked between day 7 and 15 pi and reached very significant levels (titer >10,000) in the IN-boosted group with protein alone as well as in the IN-boosted group with the adjuvanted vaccine (Figure 6).

**Figure 7:**
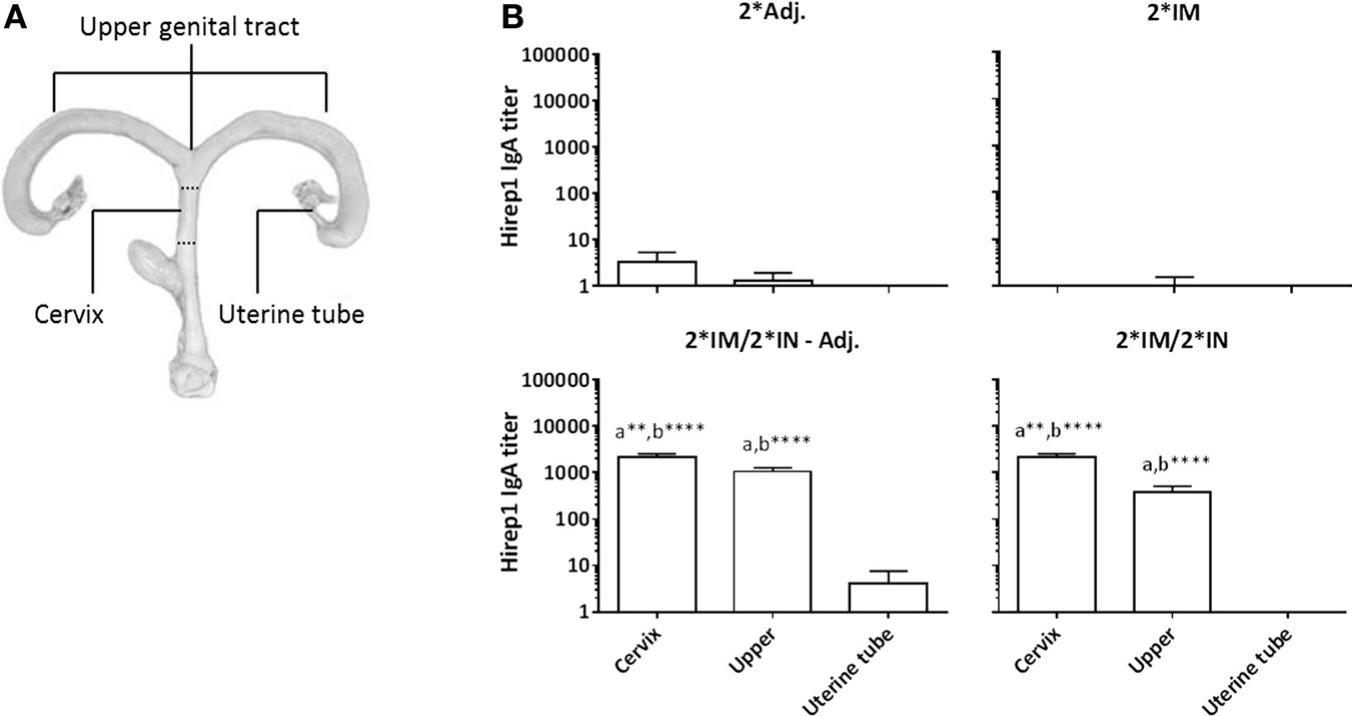
**Hirep1-specific IgA antibody responses in the genital tract on day 15 pi**. Following the immunizations as illustrated in Figure 1, minipigs were challenged vaginally with *C. trachomatis* SvD and 15 days pi, the minipigs were euthanized and swabs from the cervix, the uterine body and horns (upper) and uterine tubes were evaluated for Hirep1-specific IgA. **(A)** The definition of the three locations in the genital tract: the cervix, the upper genital tract including swabs from the uterine body and horns, and then the uterine tubes (Fallopian tubes) **(B)** The IgA titers in the four groups. Bars show mean ± SEM. Statistics: Dunn’s multiple comparisons. Symbol (a) indicates significance compared to the 2*Adj. group and (b) indicates significance compared to the 2*IM group. Asterisks indicate significance level (**P < 0.01 and ****P < 0.0001).

At the necropsy (day 15 pi), swab samples were collected from all compartments of the genital tract to evaluate the presence of antibodies on the genital mucosa. We found significant levels of specific IgA in both the cervix and the upper genital tract (uterine body and horns) in the IN-boosted groups (Figure 7A). Although not statistically significant, we also found an IgA response in the uterine tubes in the 2*IM/2*IN-Adj. group (Figure 7A). Furthermore, vaginal IgG titers were significantly increased throughout the genital tract at day 15 pi in all three vaccinated groups (data not shown).

**Figure 8:**
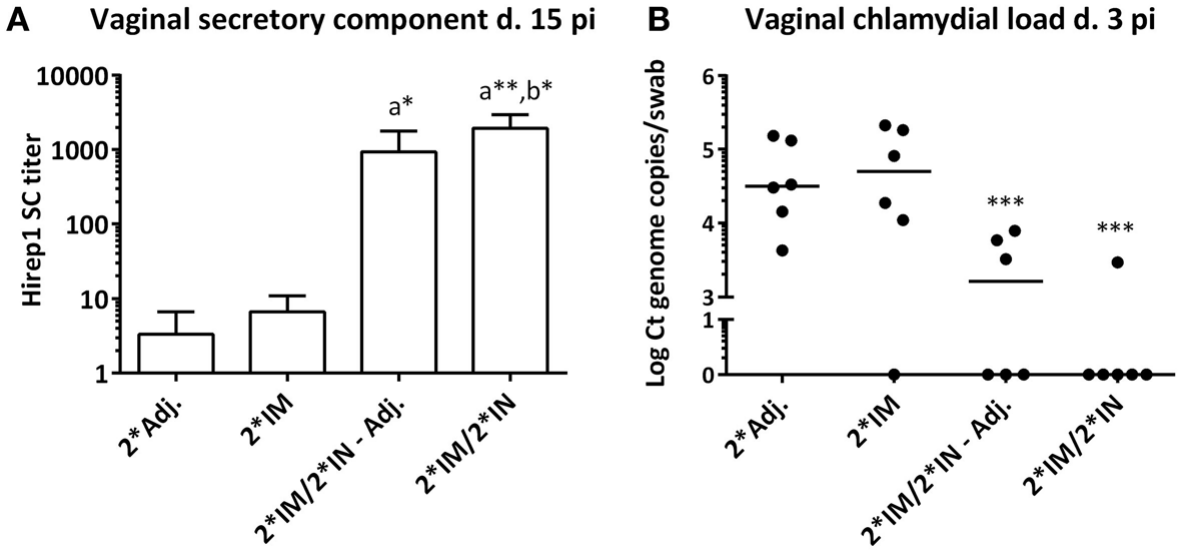
**Secretory component and chlamydial load on the vaginal mucosa following genital challenge**. **(A)** Hirep1-specific secretory component (SC) detected by ELISA in vaginal swabs on day 15 pi. Bars show mean ± SEM. **(B)** Vaginal *Chlamydia* load on day 3 pi detected by 16s qPCR in vaginal swabs. Each dot represents the mean of triplicates. A value of 0 on the *y* axis corresponds to a non-detectable value. Horizontal line represents median. Statistics: Dunn’s multiple comparison tests. Symbol (a) indicates significance compared to the 2*Adj. group and (b) indicates significance compared to the 2*IM group. Asterisks indicate significance level.

Enzyme linked immunosorbent assay detection of SC on the vaginal mucosa was performed to investigate the level of locally produced IgA. The IN-boosted groups showed a significant Hirep1 specific SC titer on day 15 pi (Figure 8A). The minipigs vaccinated only 2*IM did not have increased SC titers (Figure 8A). When comparing the vaginal IgA titers with the vaginal SC titers on day 15 pi, we found a significant correlation between the IgA and SC response against both vaccine antigens (Spearman’s correlation test; Hirep1: *r* = 0.59** and CTH93: *r* = 0.49*).

Evaluation of CMI responses at day 15 pi was performed by measuring the IFN-γ and IL-17A levels in the supernatants of lymph node cells (*Lnn. Iliosacrales)* re-stimulated with Hirep1. All three vaccinated groups showed an IFN-γ and IL-17A response in the iliosacral lymph node compared to the response in the 2*Adj. control group, although not all responses reached significant levels due to individual variation and the relatively small group sizes (Table 1).

**Table 1:** **Lymph node IFN-γ and IL-17A response to Hirep1 re-stimulation at day 15 pi**.

	IFN-γ (ng/ml)	IL-17A (pg/ml)
2*Adj.	0.15 ± 0.05	1.5 ± 0.74
2*IM	0.67 ± 0.20	20.9 ± 5.80**
2*IM/2*IN-Adj.	1.81 ± 0.66**	39.1 ± 10.7***
2*IM/2*IN	1.20 ± 0.43	69.3 ± 36.2

Data: mean ± SEM. Asterisks indicate significance level when compared to the 2*Adj. control group. Statistics: Bonferroni corrected multiple comparisons on log transformed data.

### Vaginal *C. trachomatis* Load Post Infection

Protective efficacy was evaluated by the vaginal chlamydial load after challenge infection. Vaginal swabs were taken at days 3, 5, 7, and 15 pi, and the level of chlamydial 16S DNA was evaluated by q-PCR. All pigs rapidly cleared the infection and vaccine-promoted protection could only be observed at day 3 pi, where the levels of chlamydial DNA were significantly (10-100-fold) decreased in the two IN-boosted groups of pigs compared to the 2*Adj. control group and the 2*IM immunized pigs (Figure 8B). There was a highly significant inverse correlation between the vaginal chlamydial load on day 3 pi and the vaginal Hirep1 specific IgA on day 5 pi (Spearman’s *r* = −0.64***), day 7 pi (Spearman’s *r* = −0.71***), and day 15 pi (Spearman’s *r* = −0.67***). Furthermore, there was a highly significant inverse correlation between the chlamydial load on day 3 pi and the levels of SC on day 15 pi (Spearman’s *r* = −0.71****).

## Discussion

It is becoming increasingly clear that mucosal IgA and IgG antibodies represent an important part of the protective immune response against genital *C. trachomatis* infection (8, 10, 11, 39, 40). In the current study, we demonstrate that an immunization strategy with IM priming and IN boosting, results in a strong significant SIgA response in the genital tract of female minipigs following genital challenge and furthermore that the local SIgA response appears to be important for an accelerated clearance of a genital *C. trachomatis* infection.

Our findings are relevant in the ongoing debate on the importance of antibodies in the protection against *C. trachomatis* infection and pathology (8, 10, 39, 41). IgA has recently attracted significant attention as an important part of the protective immunity against *C. trachomatis* (10, 40), and SIgA may be of particular relevance to protection against *C. trachomatis* due to its neutralizing and anti-inflammatory capacities, compared to monomeric IgA and IgG (16). IgA produced by local plasma cells in the genital mucosa will predominantly be of the polymeric forms and therefore be attached to the SC, when transported across the genital epithelium via the polymeric immunoglobulin receptor (pIgR) (16, 18, 20, 42, 43). Hence, the SC we detected on the vaginal mucosa will most likely represent locally produced IgA. We cannot rule out that the small amounts of serum polymeric IgA (pIgA) could be transported across the epithelium with the pIgR, and therefore attached to SC. However, since we did not detect any specific IgA response in serum in the IN-boosted groups, our data suggest that the vaginal pIgA is locally produced. Whether IgA-committed B cells are found in the genital tract already after IN immunizations and just need to be activated in the presence of antigen during infection, or whether IgA-committed B cells needs to migrate into the genital tract mucosa after challenge infection is currently unknown.

Earlier vaccine trials in pigs, with IN vaccination, have not shown a mucosal immune response in the genital tract (44) and it has never been experimentally addressed whether the nasal–genital immunization route exists in pigs. The significant genital IgA response after infection in the IN-boosted groups, in this study, confirms that the nasal–genital bridge does exist in pigs and our data thereby demonstrate the importance of a prime-boost strategy for the induction of local genital IgA. Our findings are in agreement with recent studies in mice, showing that parental priming followed by mucosal boosting efficiently recruits CD4 T cells to the mucosa and facilitates IgA secretion (1, 45). Our study is the first demonstration of vaccine-promoted IgA in the female genital tract in a more human-relevant porcine model, confirming the findings from mice that IN booster immunizations can induce genital IgA (1).

IN-boosted pigs were significantly protected at day 3 pi compared to the IM vaccinated and whereas these immunization strategies promoted identical serum IgG levels they clearly differed in local vaginal IgA levels. The highly significant inverse correlation between the vaginal IgA SC response and the chlamydial load therefore suggest that IgA in the minipig model is involved in protection against *C. trachomatis*. It is however important to emphasize that our data do not exclude a role for IgG in protection against *C. trachomatis*. Another recent study in the minipig model with the same vaccine antigen suggests that IM vaccination (without mucosal boost), can confer protection even without IgA, if the IgG titers are sufficiently high (33). Thus both vaginal IgG and IgA seem to have protective potential in the porcine model.

In the present study, the infection was rapidly eliminated, and no difference in vaginal chlamydial load was detected beyond day 3 pi, which limits the ability of this model for the study of, e.g., the influence of Th1 cells on the more chronic stages of infection. The reason for this fast clearance may be related to, i.e., the inoculation during estrus as reported for other pathogens (46); a possibility that we are currently investigating (data not shown).

The CAF01 adjuvant used in this study signals through the CLEC receptor Mincle and induces a Th1/Th17 response together with high antibody titers in mice (29, 47). In this study, we show that the IM immunizations with the CAF01 adjuvanted vaccine induce high titers of neutralizing IgG and a Th1/Th17 response in pigs. The Th1/Th17 profile of this adjuvant may be a very good match for a *C. trachomatis* vaccine. IFN-γ (Th1) is generally accepted as a key cytokine for protection against *C. trachomatis* and activates a number of important effector pathways of relevance for elimination of the intracellular bacteria (41, 48). Th17 cells on the other hand, have the potential to transform into follicular helper T (Tfh) cells with the ability to induce IgA-isotype switched B cells (26) and recently it was shown that IL-17 is necessary for the induction of IgA in the airway mucosa (Christensen et al., unpublished). Furthermore, IL-17 facilitates B cell recruitment and upregulation of the pIgR in the airways (25).

Our study compared IN boost with or without adjuvant and found similar significant levels of nasal and genital IgA in both of the IN-boosted groups, suggesting that in minipigs primed by a CAF01 adjuvanted vaccine, the presence of adjuvant in the boost may not be critically important. Thus although CAF01 has been described to function as a mucosal adjuvant (49), its effect on IgA induction when administered as a mucosal booster on top of a strong systemic response is limited. Whether this lack of an additive value of mucosal adjuvant in Th1/Th17 primed animals is a general phenomenon is not clear. It is likely that using stronger experimental mucosal adjuvants like cholera toxin could improve the effect of nasal boosting even further also in primed animals.

For the purpose of human trials and safety issues regarding IN administration of vaccines, it is promising that the antigen alone is capable of inducing a mucosal immune response similar to the IN vaccine with CAF01.

In conclusion, our data show that IN booster immunizations can induce a significant SIgA response in the genital tract of female minipigs and suggest that this response is involved in the accelerated clearance of a genital *C. trachomatis* infection. This information is important both for our understanding of protective immunity and future vaccine strategies against this important pathogen.

## Author Contributions

EL made the inoculum, performed the experiment, laboratory analysis, statistics, interpreted data, and drafted the figures and manuscript. FF and PA planned the study, interpreted data, and revised figures and the manuscript. SB planned the study and performed some vaccinations. KE, AO, JA, and GJ planned the study. All authors approved the final manuscript.

## Conflict of Interest Statement

The authors declare that the research was conducted in the absence of any commercial or financial relationships that could be construed as a potential conflict of interest. Peter Andersen, Anja Weinreich Olsen, and Frank Follmann are co-inventors on a patent application relating to *C. trachomatis* vaccines. All rights have been assigned to Statens Serum Institut, a Danish not-for-profit governmental institute.

## Abbreviations

CMI, cell-mediated immune; Ct, SvD *Chlamydia trachomatis* serovar D; DEPC, diethyl dicarbonate; EB, elementary body; ELISA, enzyme linked immunosorbent assay; F, forward; IC, internal control; IFN-γ, interferon gamma; IFU, inclusion forming unit; IL-17A, interleukin 17A; IM, intramuscular; IN, intranasal; pi, post infection; pIgR, polymeric immunoglobulin receptor; R, reverse; SIgA, secretory IgA; SvD, serovar D.
